# Clonal dissemination of *Staphylococcus aureus* isolates causing nosocomial infections, Tehran, Iran

**DOI:** 10.22038/ijbms.2018.30067.7245

**Published:** 2019-03

**Authors:** Mehdi Goudarzi, Gita Eslami, Razieh Rezaee, Mohsen Heidary, Saeed Khoshnood, Raheleh Sajadi Nia

**Affiliations:** 1 Department of Microbiology, School of Medicine, Shahid Beheshti University of Medical Sciences, Tehran, Iran; 2 Microbiology Department, Faculty of Biological Sciences, Shahid Beheshti University, Tehran, Iran; 3 Department of Medical Microbiology, Faculty of Medicine, Iran University of Medical Sciences, Tehran, Iran; 4 Department of Microbiology, Faculty of Medicine, Ahvaz Jundishapur University of Medical Sciences, Ahvaz, Iran; 5 Student Research Committee, Ahvaz Jundishapur University of Medical Sciences, Ahvaz, Iran

**Keywords:** Agr, MLST, MRSA, SCCmec, Spa, Staphylococcus aureus

## Abstract

**Objective(s):**

In the current research, the prevalence of *Staphylococcus aureus* clones and genes encoding antimicrobial resistance and toxins were examined among 120 *S. aureus* strains from nosocomial infections in tehran, Iran.

**Materials and Methods:**

Antimicrobial susceptibility was examined, based on disk diffusion and PCR method to identify resistance and toxin-encoding genes. Based on the polymorphisms in SCC*mec*, *agr, spa*, and MLST, the isolates were typed.

**Results:**

Among 120 *S. aureus *isolates, 85 (70.8%) were methicilin resistant *S.* *aureus (*MRSA), and 35 (29.2%) were methicilin sensetive *S. aureus (*MSSA). The tested isolates contained resistance genes, including *ant(4΄)-Ia* (90%), *aac(6΄)-Ie/aph(2˝)* (80%), *aph(3΄)-IIIa* (30%), *erm(A)* (26.7%), *erm(B)* (10.8%), *erm(C)* (11.7%), *msr(A)* (40.8%), *msr(B) *(14.2%), *tet(M)* (45.8%), and *mupA* (8.3%). The MRSA strains were clustered into six different clones. The most common genotypes included ST239-SCC*mec* III/t037 (23.3%), ST239-SCC*mec* III/t388 (22.5%), ST22-SCC*mec* IV/t790 (8.3%), ST15-SCC*mec* IV/t084 (7.5%), ST585-SCC*mec* III/t713 (5%), and ST239-SCC*mec* III/t924 (4.2%), respectively. ST182/t196 (8.3%) and ST123/t171 (5%) belonged exclusively to MSSA strains. Overall, 10 (66.7%) and 5 (33.3%) out of 15 isolates with *pvl* genes were attributed to clones ST22-SCC*mec* IV/t790 and ST15-SCC*mec *IV/t084, respectively. ST22-SCC*mec* IV/t790, ST239-SCC*mec* III/t037, and ST15-SCC*mec* IV/t084, were related to high-level mupirocin-resistant phenotypes.

**Conclusion:**

The genetic diversity of *S. aureus* was confirmed in our hospitals, and ST239-SCCmec III/t037 showed a relatively high prevalence in our study. It seems that assessment of resistance and virulence genes in different *S. aureus* molecular types is necessary for proper antibiotic consumption.

## Introduction

*Staphylococcus aureus, *which is described as a common nosocomial pathogen, is responsible for various diseases, such as food poisoning, osteomyelitis, wound infections, and even fatal conditions, such as endocarditis (1). Over the past few decades, it has been well-documented that the pathogen’s resistance potential to antimicrobial agents, especially methicillin, may lead to its persistence in the hospital and community (2). In 1961, the first case of methicillin-resistant *S. aureus* (MRSA) occurred in the UK (3). The prevalence of this infection has steadily increased since then, as confirmed in several studies, raising major concerns about the global increase in its prevalence, as well as its associated mortality and morbidity in the healthcare setting, especially intensive care units (ICUs) due to MRSA infections (1-3).

Resistance to methicillin is attributed to β-lactamase expression or changes in the structure of *mecA* gene-encoded penicillin-binding protein-2. Generally, *mecA* gene (21-67 kbp) is recognized as a staphylococcal cassette chromosome *mec* (SCC*mec*). Generally, SCC*mec* is categorized into 11 types with respect to *mec* genes and *ccr* gene complexes (4). Identification of SCC*mec* type among *S. aureus* clinical isolates can be useful in molecular typing of MRSA strains (5).

Based on previous findings, SCC*mec* I-III and IV-V are respectively responsible for the most common hospital-acquired and community-acquired MRSA (HA-MRSA and CA-MRSA, respectively) infections. HA- and CA-MRSA strains can be distinguished with respect to some genotypic, phenotypic, and epidemiological characteristics, as well as virulence factors (4, 5).

The emergence and prevalence of MRSA infections containing multidrug-resistant (MDR) genes have significantly limited the availability of antibiotics over the past decades. In addition, the growing emergence of MDR-MRSA strains poses a major global health concern (1). Wide resistance to β-lactams, besides other antibiotics, including aminoglycosides, lincosamides, and macrolides, has been shown in MRSA strains (6).

Aminoglycosides play a key role in serious anti-staphylococcal therapies. According to the previous researches, resistance to aminoglycosides is attributed to aminoglycoside-modifying enzymes (AMEs) including aminoglycoside nucleotidyltransferases, aminoglycoside phosphotransferases and aminoglycoside acetyltransferases (7). *mupA *and *mupB* genes are responsible for resistance to mupirocin which is used to treat various types of skin diseases caused by *S. aureus* (8). Resistance to macrolides as protein synthesis inhibitors is mediated by *msr *genes activating efflux pumps and* erm *genes modifying the ribosomal binding site (9). Thus, in spite of new antibiotics introduction, concerning the emergence and dissemination of antibiotic resistance genes, MRSA infections treatment is still a great dilemma worldwide.

This study was conducted to identify antibiotic resistance patterns and the carriage of resistance and virulence genes as well as major MRSA clones by MLST,* spa*, SCC*mec* and *agr *techniques in clinical samples taken from patients in Tehran, Iran.

## Materials and Methods

***Sampling, MRSA isolation and antibacterial susceptibility testing***

This cross-sectional study included 368 clinical samples from wound, blood, and urine specimens during April-December 2016. Ethics Committee of Shahid Beheshti University of Medical Sciences approved the implementation of this study (IR.SBMU.SM.REC.1395.157). All patients signed written informed consent forms.

After the rapid transfer of the specimens to the laboratory, *S. aureus* identification was performed, based on the conventional biochemical tests. *S. aureus* identification was confirmed based on the PCR assay for *nucA* gene (10). According to the Clinical and Laboratory Standards Institute (CLSI) standards, resistance to methicillin was examined with oxacillin and cefoxitin (1 and 30 µg, respectively) disks in Mueller-Hinton agar plates (Merck; Germany) containing 4% sodium chloride (11). For further molecular analysis, confirmed isolates were stored in Tryptic Soy Broth with 15% glycerol (Merck; Germany) at a temperature of -70 ^°^C. Afterwards, based on the Kirby-Bauer method, the susceptibility profiles to 12 antibiotics including tetracycline (T 30 µg), clindamycin (CD 2 µg), ciprofloxacin (CIP 5 µg), trimethoprim- sulfamethoxazole (TS 2.5 µg), kanamycin (K 30 µg), ceftriaxon (CRO 30 µg), quinupristin-dalfopristin (SYN 15 µg), erythromycin (E 15 µg), amikacin (AK 30 µg), gentamicin (GM 10 µg), tobramycin (TN 10 µg), teicoplanin (TEC 30 µg), penicillin (PG 10 µg), and linezolid (LZD 30 µg) (Mast, UK) were determined, based on the CLSI criteria (11).

Using E-test strips (bioMe´rieux), the minimum inhibitory concentrations (MICs) were measured for mupirocin and vancomycin. Resistance to three antibiotic groups or more, besides beta-lactams, was defined as MDR. High mupirocin resistance was defined as antibiotic use ≥ 256 mg/l. Growth in a well containing clindamycin and erythromycin (0.5 and 4 μg/ml, respectively) indicated inducible macrolide-lincosamide-streptogramin B and/or clindamycin resistance phenotypes; otherwise, constitutive MLSB and/or clindamycin resistance phenotype was confirmed (11). ATCC29213 and ATCC25923 (*S. aureus*) were considered as the reference strains for the quality control purposes.

***Extraction of genomic DNA***

For extracting genomic DNA, pure overnight *S. aureus* cultures were used on 5% sheep blood agar (BA; Merck, Germany), based on the protocols of InstaGene Matrix kit (BioRad, USA). 

***Resistance and toxin genes profiling***

To identify toxin (*etb*, *tst, pvl*, *eta*) and resistance (*tet(M), aac (6΄)-Ie/aph (2˝), mupA, erm(A), msr(A), msr(B), erm(B), erm(C), ant (4΄)-Ia, aph (3΄)-IIIa*) genes, PCR assay was carried out. The details of the degenerated primers in this study are described in Table 1.

***Multiplex PCR for SCCmec typing***

According to a study by Boy and colleagues, for SCC*mec* typing, multiplex PCR amplification was performed with specific primers (4). The controls comprised of the MRSA strains, i.e., ATCC 10442, N315, 85/2082, MW2, and WIS (attributed to types I, II, III, IV, and V, respectively).

***Multiplex PCR amplification for agr typing***

In addition, for *agr* typing, multiplex PCR amplification was carried out, using forward (Pan) and reverse (agr1 to agr4) primers for the *agr* groups as previously recommended by Gilot* et al* (18). The specific oligonucleotide primers are listed in Table 1.

***spa typing***

On the other hand, *spa* gene was detected according to a study by Harmsen and colleagues (19). After the positive *spa* PCR products were purified, DNA sequencing was carried out in both strands (Macrogen; South Korea). Chromas 1.45 (Australia) was used to edit the sequences. To assign the sequences to specific *spa* types, the Ridom SpaServer database was searched.

***MLST technique***

Via amplification and sequencing, MLST was carried out on *S. aureus* isolates. The internal fragments of housekeeping genes were used to identify the allelic profiles; these genes included *gmk, arcC, aroE, glpF, pta,* *yqiL, *and* tpi*. The isolate was assigned a sequence type (ST) after comparing the sequences with the *S. aureus *MLST database.

## Results

***Sampling and antibiotic susceptibility***

In this study, out of 368 samples obtained from various clinical specimens, 120 isolates (83 (69.2%) obtained from men and 37 (30.8%) from women) were identified as *S. aureus. *These isolates originated from wound (60%), blood (20.8%) and urinary tract infections (19.2%). Of the 120 *S. aureus* clinical isolates obtained from the hospitalized patients, 85 (70.8%) were MRSA and 35 (29.2%) were methicillin susceptible *S. aureus* (MSSA). 

None of the isolates were susceptible to all of the antimicrobial agents tested regarding* in vitro* antimicrobial susceptibility tests. All isolates were susceptible to vancomycin, among which 55 (45.8%), 48 (40%) and 17 (14.2%) isolates had a MIC of 0.5, 1 and 2 µg/ml, respectively. 

Among the 35 MSSA isolates, no mupirocin resistance was detected, whereas 30 MRSA isolates (35.3%) were mupirocin resistant. Of these mupirocin resistant isolates, 14 (46.7%) and 16 (53.3%) had high and low resistance levels, respectively. All the high-level mupirocin-resistant (HLMUPR) isolates were collected from wound samples. Patients diagnosed with mupirocin-susceptible and -resistant MRSA infections were not significantly different in terms of age and gender (*P*= 0.145 and 0.128, respectively). The frequency of resistance for MRSA and MSSA isolates to different antibacterial agents are presented in Table 2. 

**Figure 1:**
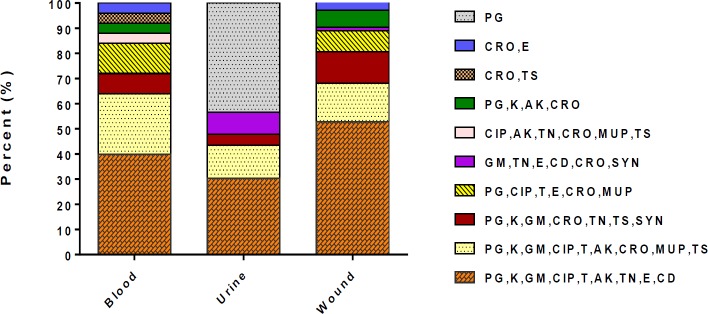
Resistance pattern of *Staphylococcus aureus* obtained from clinical samples

Of the 120 *S. aureus* isolates, 87.5% (105/120) were defined as MDR. The predominant multiple drug resistance profile among the MDR isolates were resistance to 9 and 7 antibiotics found in 75 (62.5%) and 12 (10%) isolates, respectively. Distribution of resistance profile and different clinical sample in S. aureus isolated from nosocomial infections are presented in Figure 1.

**Figure 2:**
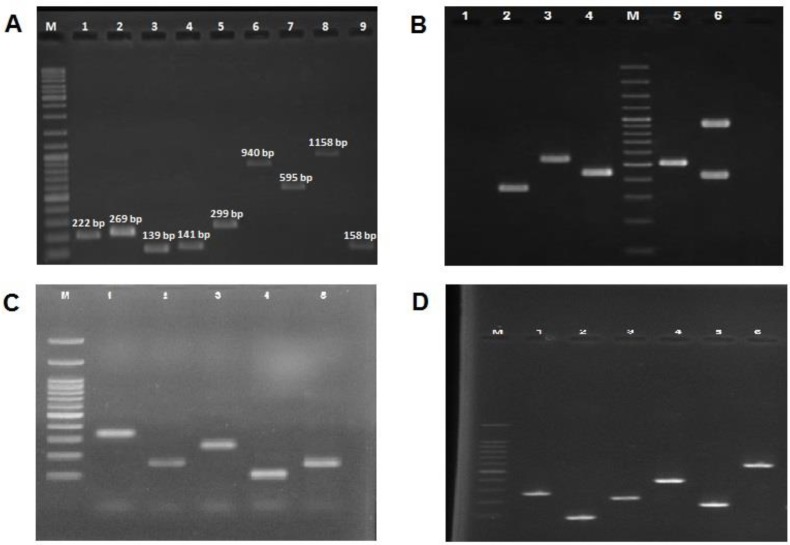
A) Lane M, 100-bp DNA Ladder (Fermentas, UK); Lane 1 PCR product of *aac(6΄)-Ie/aph(2˝) *encoding gene, Lane 2 PCR product of PCR product of aph(3΄)-IIIa encoding gene, Lane 3 PCR product of *erm(A)* encoding gene, Lane 4 PCR product of *erm(B)* encoding gene, Lane 5 PCR product of *erm(C)* encoding gene, Lane 6 PCR product of *msr(A)* encoding gene, Lane 7 PCR product of *msr(B) *encoding gene, Lane 8 PCR product of *mupA* encoding gene, and Lane 9 PCR product of *tet(M)* encoding gene. B) Lane M, DNA Ladder; lane 1 negative control, Lane 2 the 323 bp PCR product of *agr* type III, lane 3 the 575 bp PCR product of *agr* type II, lane 4 the 441 bp PCR product of *agr* type I, lane 5 the 518 bp PCR product of SCC*mec* Type III, lane 6 the 937 and 415 bp PCR products of SCC*mec *Type IV. C) Lane M, DNA ladder; lane 1-5, variable PCR product of *spa*. D) Lane M, DNA Ladder; Lane 1the 270 bp PCR product of *nucA* gene, Lane 2the 93 bp PCR product of *eta* gene, Lane 3the 226 bp PCR product of *etb* gene, Lane 4the 398 bp PCR product *oftst-1 *gene, Lane 5the 180 bp PCR product of *luk-PV* gene, Lane 6the 583 bp PCR product of *mecA* gene

**Figure 3:**
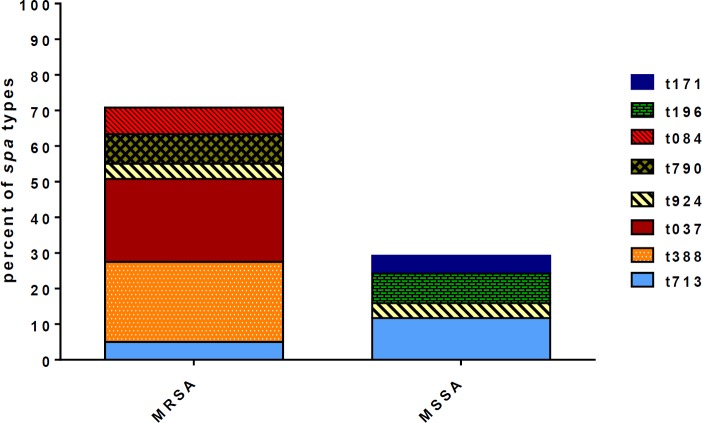
Distribution of spa types in methicilin resistant S. *aureus (*MRSA) and methicilin sensetive *S. aureus (*MSSA) strains isolated from nosocomial infections

**Figure 4:**
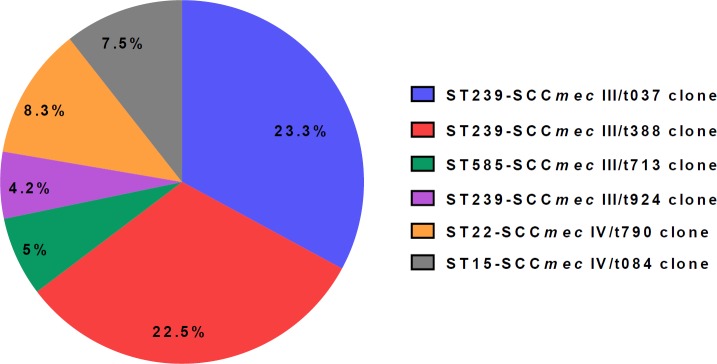
Distribution of molecular types in 85 methicilin resistant *S.* *aureus *(MRSA) strains isolated from nosocomial infections

**Table 1:** Primer and oligo sequences used in present research

Gene	Primer	Primer sequence (5´ 3´)	Length (bp)	Reference
*mecA*	F	AGA AGA TGG TAT GTG GAA GTT AG	583	(10)
	R	ATG TAT GTG CGA TTG TAT TGC		
*tst-1*	F	TTA TCG TAA GCC CTT TGT TG	398	(10)
	R	TAA AGG TAG TTC TAT TGG AGT AGG		
*nucA*	F	GCG ATT GAT GGT GAT ACG GTT	270	(12)
	R	AGC CAA GCC TTG ACG AAC TAA AGC		
*luk-PV*	F	TTC ACT ATT TGT AAA AGT GTC AGA CCC ACT	180	(12)
	R	TAC TAA TGA ATT TTT TTA TCG TAA GCC CTT		
*etb*	F	ACA AGC AAA AGA ATA CAG CG	226	(13)
	R	GTT TTT GGC TGC TTC TCT TG		
*eta*	F	GCA GGT GTT GAT TTA GCA TT	93	(13)
	R	AGA TGT CCC TAT TTT TGC TG		
*tet(M)*	F	AGT GGA GCG ATT ACA GAA	158	(13)
	R	CAT ATG TCC TGG CGT GTC TA		
*aph(3΄)-IIIa*	F	CTT GAT CGA AAA ATA CCG CTG C	269	(14)
	R	TCA TAC TCT TCC GAG CAA A		
*ant(4΄)-Ia*	F	AAT CGG TAG AAG CCC AA	135	(14)
	R	GCA CCT GCC ATT GCT A		
*aac(6΄)-Ie/aph(2˝)*	F	CCA AGA GCA ATA AGG GCA TAC C	222	(14)
	R	CAC ACT ATC ATA ACC ACT		
*erm(B)*	F	CTA TCT GAT TGT TGA AGA AGC ATT	141	(15)
	R	GTT TAC TCT TGG TTT AGG ATC AAA		
*erm(A)*	F	TAT CTT ATC GTT GAG AAG GGA TT	139	(15)
	R	CTA CAC TTG GCT GAT GAA A		
*erm(C)*	F	AAT CGT CAA TTC CTG CAT GT	299	(16)
	R	TAA TCG TGG AAT ACG GGT TTG		
*msr(B)*	F	TAT GAT ATC CAT AAT AAT TAT CCA ATC	595	(16)
	R	AAG TTA TAT CAT GAA TAG ATT GTC CTG TT		
*msr(A)*	F	GGC ACA ATA AGA GTG TTT AAA GG	940	(16)
	R	AAG TTA TAT CAT GAA TAG ATT GTC CTG TT		
*mupA*	F	CCC ATG GCT TAC CAG TTG A	1158	(17)
	R	CCA TGG AGC ACT ATC CGA		

**Table 2:** The frequency of antimicrobial resistance in 120 *S. aureus* isolates obtained from clinical specimens

Antibiotics	MSSA (n=35) n (%)		MRSA (n=85) n (%)		All (n=120) n (%)	
	S	R	S	R	S	R
penicillin	2(5.7)	33(94.3)	6(7.1)	79(92.9)	8(6.7)	112(93.3)
kanamycin	17(48.6)	18(51.4)	10(11.8)	75(88.2)	27(22.5)	93(77.5)
gentamicin	21(60)	14(40)	9(10.6)	76(89.4)	30(25)	90(75)
ciprofloxacin	23(65.7)	12(34.3)	12(14.1)	73(85.9)	35(29.2)	85(70.8)
tetracycline	21(60)	14(40)	15(17.6)	70(82.4)	36(30)	84(70)
amikacin	27(77.1)	8(22.9)	11(12.9)	74(87.1)	38(31.7)	82(68.3)
tobramycin	29(82.9)	6(17.1)	20(23.5)	65(76.5)	49(40.8)	71(59.2)
erythromycin	29(82.9)	6(17.1)	21(24.7)	64(75.3)	50(41.7)	70(58.3)
clindamycin	30(85.7)	5(14.3)	32(37.6)	53(62.4)	62(51.7)	58(48.3)
ceftriaxone	30(85.7)	5(14.3)	35(41.2)	50(58.8)	65(54.2)	55(45.8)
trimetoprim-sulfamethoxazole	33(94.3)	2(5.7)	53(62.4)	32(37.6)	86(71.7)	34(28.3)
mupirocin	35(100)	0(0)	55(64.7)	30(35.3)	90(75)	30(25)
quinupristin-dalfopristin	31(88.6)	4(11.4)	74(87.1)	11(12.9)	105(87.5)	15(12.5)
linzolid	35(100)	0(0)	85(100)	0(0)	120(100)	0(0)
teicoplanin	35(100)	0(0)	85(100)	0(0)	120(100)	0(0)
vancomycin	35(100)	0(0)	85(100)	0(0)	120(100)	0(0)

**Table 3:** Distribution of MRSA molecular types isolated from nosocomial infections

Molecular types	*agr *class	Type of toxin (No;%)	Genotypic resistance patterns (No;%)	Phenotypic resistance patterns (No;%)	No (%)
ST239- SCC*mec*III/t037	I	*tst *(15;53.6), *eta *(2;7.1)	*mecA *(28;100)*, aph(3΄)-IIIa *(20;71.4),* aac(6΄)Ie/aph (2˝)*(28;100),*erm(A) *(9;32.1),*erm(B)* (4;14.3), *erm(C)* (8;28.6), *msr(A) *(20;71.4), *msr(B) (*4;14.3)*, ant(4΄)-Ia *(28;100), *tet(M) *(28;100)	PG, GM, K, CIP, AK, T, TN, E, CD (18;64.3)	28 (23.3)
				PG, GM, K, CIP, AK, T, CRO, MUP, TS (8;28.6)	
				GM, TN, E, CD, CRO, SYN (2;7.1)	
ST239- SCC*mec*III/t388	I	*tst *(11;40.7)	*mecA *(27;100)*, aph(3΄)-IIIa *(10;37), *erm(A) *(12; 44.4),*erm(B)* (8;29.6), *msr(A) *(12;44.4), *aac(6΄)-Ie/aph(2˝) *(27;100),*msr(B) *(8;29.6)*, ant(4΄)-Ia *(27;100),*tet(M) *(12;44.4)	PG, K, CIP, GM, AK, T, CD, TN, E (20;74.1)	27 (22.5)
				PG, E, CIP, CRO, T, MUP (5;18.5)	
				PG, K, AK, CRO (2;7.4)	
ST585- SCC*mec*III/t713	I	*tst *(6;100), *etb *(2;7.7)	*mecA* (6;100)*, aph(3΄)-IIIa *(1;16.7), *erm(A) *(3;50), *ant(4΄)-Ia *(6;100), *erm(C)* (5;83.3), *msr(A) *(5;83.3),* aac(6΄)-Ie/aph(2˝) *(6;100),	PG, K, CIP, GM, AK, T, CD, TN, E (2;17.9)	6 (5)
				PG, TS, K, TN, GM, CRO, SYN (4;35.7)	
ST239- SCC*mec*III/t924	III	*tst *(5;100)	*aph(3΄)-IIIa*(5;100), *mecA *(5;100)*, aac(6΄)-Ie/aph(2˝) *(5;100), *erm(A) *(5; 100), *erm(B)* (1;20),* ant(4΄)-Ia *(5;100),	PG, CD, K, CIP, GM, AK,T, TN, E (1;20)	5 (4.2)
				PG, TS, K, GM, TN, CRO, SYN (3;60)	
				CIP, AK, TN, CRO, MUP, TS (1,20)	
ST22-SCC*mec*IV/t790	I	*pvl *(10;100), *tst *(3;30), *eta *(3;30), *etb *(1;10)	*mecA *(10;100)*, erm(A) *(3;30), *erm(C)* (1;10), *msr(A) *(9;90), *msr(B) *(5;50)*, ant(4΄)-Ia *(10;100), *tet(M) *(10;100)*, aac(6΄)-Ie/aph(2˝) *(10;100), *mupA *(6;60)	PG, MUP, GM, AK, CIP, T, K, CRO, TS (5;50)	10 (8.3)
				GM, TN, E, CD, CRO, SYN (1;10)	
				PG, K, AK, CRO (1;10)	
				PG, CIP, T, E, CRO, MUP (3;30)	
ST15- SCC*mec*IV/t084	II	*pvl *(5;55.6), *tst *(8;88.9), *eta *(4;44.4)	*mecA*(9;100)*, aac(6΄)-Ie/aph(2˝)*(9;100), *ant(4΄)-Ia*(9;100), *msr(A) *(3;33.3), *tet(M) *(5;55.6)*, mupA *(4;44.4)	PG, TN, K, CD, GM, CIP, T, AK, E (2;22.2)	9 (7.5)
				PG, CIP, T, E, CRO, MUP (1;11.1)	
				PG, CRO, K, AK, GM, CIP, T, MUP, TS (6;66.7)	

***The distribution of resistance genes***

In addition, the frequency of antibiotic resistance genes was measured. The genes included *ant(4΄)-Ia* (90%), *aac(6΄)-Ie/aph(2˝)* (80%), *aph(3΄)-IIIa* (30%), *erm(A)* (26.7%), *erm(B)* (10.8%), *erm(C)* (11.7%), *msr(A)* (40.8%), *msr(B)* (14.2%), *tet(M) *(45.8%), and *mupA* (8.3%) (Figure 2). The MRSA strains contained *ant(4΄)-Ia, aac(6΄)-Ie/aph(2˝)*, and *mecA* genes. Other detected antibiotic resistance genes included *tet(M)* (64.7%), *msr(A)* (57.6%), *aph(3΄)-IIIa *(42.3%), *erm(A)* (37.6%), *msr(B)* (20%), *erm(C)* (16.5%), *erm(B)* (15.3%), and *mupA *(11.8%), while 31.4% and 56.7% of MSSA strains were found to respectively carry *aac(6΄)-Ie/aph(2˝)* and *ant(4΄)-Ia*. Particularly, the MRSA strains contained more resistance genes, compared to the MSSA isolates.

Fourteen (11.7%) out of 30 mupirocin-resistance *S. aureus* isolates were identified as HLMUPR-MRSA, while *mupA* gene was confirmed in 10 isolates (71.4%). In addition, *ant(4΄)-Ia* (108, 90%), followed by *aac(6΄)-Ie/aph(2˝)* (96, 80%), was recognized as the most common aminoglycoside resistance gene. A total of 75 (62.5%) isolates contained *ant(4΄)-Ia*, as well as *aac(6΄)-Ie/aph(2˝)*. On the other hand, 15 (12.5%) isolates harbored *aph(3΄)-IIIa*, besides *ant(4΄)-Ia*. Also, eleven (9.2%) isolates harbored *ant(4΄)-Ia, aph(3΄)-IIIa*, and *aac(6΄)-Ie/aph(2˝)* genes, while 10 (8.3%) contained *aac(6΄)-Ie/aph(2˝)*, as well as *aph(3΄)-IIIa*; however, *ant(4΄)-Ia *alone was confirmed in 7 (5.8%) strains. Resistance to tetracycline was observed among 84 (70%) *S. aureus* isolates, 55 (45.8%) of which harbored *tet(M)* gene. Antibiotic resistance genes showed the highest prevalence among MRSA strains from wound infections.

***Virulence gene profiling***

Among toxin-encoding genes, the highest and lowest frequencies were attributed to *tst* (58; 48.3%) and *etb* (3; 2.5%) genes, respectively (Figure 2). In present work, 12.5% of the isolates were positive for *pvl* gene and *eta* gene was identified in 7.5% of the isolates. The only toxin encoding gene among the MSSA isolates was *tst* gene (10 out of 120 isolates, 8.3%). *S. aureus *isolates harbouring *tst* gene were isolated from wound (32; 55.2%), blood (17; 29.3%), and urinary tract infections (9; 15.5%) while *pvl* positive isolates were detected in wound (12; 80%) and blood (3; 20%) samples. Eight isolates carrying *pvl* and *tst* genes, simultaneously, had *mupA* gene.

*Distribution of SCCmec types*

According to SCCmec typing, 66 (77.6%) and 19 (22.4%) MRSA isolates contained SCCmec types III and IV, respectively. No isolate harbored SCCmec type V, II, or I. Based on the multiplex PCR, isolates positive for PVL were attributed to SCCmec type IV, while tst gene was found in MRSA isolates from SCCmec III and IV. The HA-MRSA origin was emphasized by the presence of SCCmec type III.

*Frequency of agr types*

agr typing indicated that type I was the predominant agr type present in 91 isolates (75.8%), followed by type III which was present in 20 isolates (16.7%). agr type II was detected in 9 isolates (7.5%). Among 35 MSSA isolates, 20 and 15 harboured agr types I and III respectively (Figure 2).

*spa typing*

spa typing was performed for all *S. aureus* isolates. spa typing discriminated eight different types: t037 (23.3%), t388 (22.5%), t713 (16.8%), t924 (8.3%), t790 (8.3%), t196 (8.3%) t084 (7.5%) and t171 (5%) (Figure 2). All the spa types except t196 and t171 were found in MRSA strains. Distribution of spa types among methicillin resistance and methicillin sensitive strains are presented in Figure 3. The most prevalent spa type among MSSA strains was t713 (11.7%, 14/120), followed by t196 (8.3, 10/120), t171 (5%, 6/120) and t924 (4.2%, 5/120), respectively.

***MLST***

Apparently, 120 isolates belonged to six different STs including ST239 (65 strains), ST22 (10 strains), ST182 (10 strains), ST15 (9 strains), ST585 (20 strains) and ST123 (6 strains). ST239, ST585, ST182 and ST123 were found in MSSA strains. It should be noted that of these STs, ST182 and ST123 belonged exclusively to MSSA strains. In conclusion, MRSA strains are clustered into six different groups. ST239-SCC*mec* III/t037 was found to be the most prominent MRSA clone identified in this study. Distribution of MRSA clones isolated from nosocomial infections are presented in Figure 4.

Fifteen PVL-carrying strains in our study belonged to ST22-SCC*mec* IV/t790 (10 isolates, 66.7%) and ST15-SCC*mec* IV/t084 (5 isolates, 33.3%) clones. Among the isolates under study, 58 (48.3%) isolates harboring *tst-1 *were distributed in ST239-SCC*mec* III/t037 (15 isolates, 25.9%), ST239-SCC*mec* III/t388 (11 isolates, 19%), ST15-SCC*mec* IV/t084 (8 isolates, 13.8%), ST585-SCC*mec* III/t713 (6 isolates, 10.3%), ST239-SCC*mec* III/t924 (5 isolates, 8.6%), and ST22-SCC*mec* IV/t790 (3 isolates, 5.2%) clones. Among examined isolates, nine isolates (7.53%) were found to carry the* eta *gene. The *eta* positive isolates were distributed in ST15-SCC*mec* IV/t084 (4 isolates, 44.4%), ST22-SCC*mec* IV/t790 (3 isolates, 33.3%) and ST239-SCC*mec* III/t037 (2 isolates, 22.2%) clones. The *etb* gene was detected in ST585-SCC*mec* III/t713 (66.7%, 2/3) and ST22-SCC*mec* IV/t790 (33.3%, 1/3) clones. MRSA clones resistance profile varied. Resistance to mupirocin was detected in all the MRSA clones with the exception of ST585-SCC*mec* III/t713 clone. Interestingly, mupirocin resistant MSSA isolates belonged to ST182/*spa* type t196. HLMUPR-MRSA strains were detected in ST22-SCC*mec* IV/t790 (42.8%, 6/14), ST15-SCC*mec* IV/t084 (28.6%, 4/14) and ST239-SCC*mec* III/t037 (28.6%, 4/14) clones. 

cMLS_B_ phenotype was detected in ST239-SCC*mec* III/t037 (43.5%, 20/46), ST239-SCC*mec* III/t388 (43.5%, 20/46), ST585-SCC*mec* III/t713 (4.3%, 2/46), ST15-SCC*mec* IV/t084 (4.3%, 2/46), ST239-SCC*mec* III/t924 (2.2%, 1/46) and ST22-SCC*mec* IV/t790 (2.2%, 1/46) while iMLS_B_ phenotype distributed among 3 major clones ST239-SCC*mec* III/t388 (55.6%, 5/9), ST22-SCC*mec* IV/t790 (33.3%, 3/9) and ST15-SCC*mec* IV/t084 (11.1%, 1/9). Of 12 MSSA isolates with cMLS_B_ phenotype, 5 isolates belonged to ST182/t196, 4 isolates belonged to ST239/t924, 2 isolates belonged to ST585/t713 and 1 isolate belonged to ST123/t171. All the iMLS_B _phenotype among MSSA strains belonged to ST585/t713. Characteristics of MRSA clones are presented in Table 3.

## Discussion

In consistent with the results of a multicenter study made by Ko *et al.* (20), a relatively high resistance to gentamicin (75%), amikacin (68.3%), kanamycin (77.5%), and tobramycin (59.2%) was reported in this study which was higher than the resistance rate reported by Goudarzi *et al* (12). In contrast to the findings of Marghaki *et al* `s study (6) who reported high frequency of *aac(6′)/aph(2′′) *gene (40.3% ) in comparison with other AME genes, in the present study, *ant(4΄)-Ia* (90%) was the most frequent AME gene in *S. aureus* isolates. However, in this study, *aph*(3*΄)-IIIa* gene frequency rate was higher than the study reported by Ida *et al.* (8.9%)(21) and Marghaki *et al* (15.7%) (6).

Mupirocin as an important agent in the control of MRSA outbreaks and eradicating MRSA colonization was used for the treatment of different types of staphylococcal skin infections. Long period widespread use of mupirocin may lead to the emergence of mupirocin resistance *S. aureus* strains (8). In this survey, 30 isolates (25%) presented mupirocin resistance phenotype and 14 (11.7%) isolates were confirmed as HLMUPR-MRSA which is relatively lower than study in Iran (40%) (22) and higher than study in India (5%) (23) and Jordan (2.6%) (24). However, as a result of proper mupirocin prescription in clinic, in our study mupirocin resistant *S. aureus* isolates were found to be lower. We detected the resistance gene *mupA* in 10 isolates (8.3%) which is lower than 12.6% (25) and 25% in Iran (22). 

In accordance with the results of our previous study (25) a high tetracycline resistance was seen in the present work (70%). In consistent with others (25), in our study the *tet(M)* gene that may cause tetracycline resistance was detected in 55 (45.8%) isolates. 

In this study, 58 isolates (48.3%) presented cMLSB phenotype while the frequency of iMLSB phenotype was 10% (12/120). In consistent with our findings, Schreckenberger *et al.* (7%) reported low frequency of iMLSB phenotype among *S. aureus* isolates as well (26). According to the literature, there are discrepant rates of inducible clindamycin resistance in different geographic area. Rashid Nezhad *et al.* performed a study in seven Iranian teaching hospitals (25). They found that the frequency of cMLSB, iMLSB and MLS_B_ phenotypes was 52.6%, 12.6%, and 5.3% respectively. Similarly, Fiebelkorn *et al.* in USA (27) reported that of 114 *S. aureus* isolates resistance to erythromycin, 34% and 29% indicated constitutive and inducible resistance pattern respectively. In a Canadian survey (28), the frequency of iMLSB and cMLSB phenotypes was found to be 64.7% and 35.3% respectively. In current work, the frequency of constitutive resistance was found to be higher than inducible resistance which is in line with the findings reported by Rashidi Nezhad* et al* (25). 

As previously mentioned, resistance to macrolides is encoded by genes often carried on plasmids (*erm(C)*) or transposons (*erm(A)* and *erm(B)*) and *msr* genes expressing active efflux pumps mainly *msr(A)*) (9). Our results revealed the frequency of *msr(A)*, *erm(A)*, *msr(B),* *erm(C)*, and* erm(B)* genes to be 40.8%, 26.7%, 14.2%, 11.7%, and 10.8% respectively. In contrast to Rashidi Nezhad^’^s study (25) who reported *erm(A)* gene as the predominant gene among the isolates with inducible phenotype and *erm(C)* among the isolates with the constitutive phenotype, our finding revealed that the *msr(A)* gene was the most common gene among strains with the constitutive phenotype (35; 29.2%), followed by *erm(A)* (21; 17.5%), *erm(C)* (11; 9.2%), *erm(B)* (10; 8.3%), *msr(B)* (10; 8.3%) while *erm(A)* (4; 3.3%), *erm(B)* (2; 1.7%), *erm(C)* (1; 0.83%), *msr(A)* (5; 4.2%) and *msr(B)* (1; 0.83%) were much more common among the isolates with inducible phenotype. It is worth mentioning that the frequency of *erm* and *msr *genes depends on the bacterial species as well as the geographic region in which the study is carried on. 

Type III was the most frequently found SCC*mec* (77.6%), according to the results of SCC*mec* typing, associated with an MDR pattern among MRSA isolates. This is in consistence with the results reported by Japoni and colleagues (10). The nosocomial origin of the samples was confirmed by the high frequency of SCC*mec* type III.

A significant relationship was found between the expression of virulence factors and specific *agr* locus. In consistence with our previous study, the most common *agr* types were* agr* type I (75.8%), type III (16.7%), and type II (7.5%), respectively. Goudarzi *et al.* (12) reported *agr* type III to be the most frequent type of SCC*mec* in Iran. According to several studies, the frequency of toxin and adhesion molecules was higher in isolates harboring *agr* type I gene in comparison with those harboring *agr* type III; this is in agreement with our study. Therefore, it can presume that regulation of staphylococcal adhesion molecules and toxins is associated with *agr* type I.

According to the *spa* typing results, *spa* t037 was recognized as the most common *spa* type (23.3%). This *spa* type was reported from Saudi Arabia, China, Iran as well as among HA-MRSA isolates found in Europe, America and other regions of Asia (12, 29, 30).

In present study *spa* type t388 was estimated at 22.5%. This *spa *type has also been reported in a study in Iran (31). Similarly, in a study performed in Taiwan, Ho *et al* described *spa* type t388 in MRSA strains recovered from blood cultures in different medical centers (32). It seems that the prevalence of t388 is progressively increased and has been successfully established in our healthcare settings. In our study, the frequency of t713 and t924, earlier reported in UAE and Iran (12), were found to be 16.8% and 8.3% respectively. 

In contrast with previous study conducted in Iran (12), in this study, low frequency of t790 and t084 *spa* types among our isolates, was also demonstrated.  For the first time we are reporting t196, and t171 *spa* types in MSSA strains from Iran.

Using various MRSA typing methods, the isolates were attributed to six different clones, namely ST239-SCC*mec* III/t388, ST22-SCC*mec* IV/t790, ST239-SCC*mec* III/t037, ST15-SCC*mec* IV/t084, ST585-SCC*mec* III/t713, and ST239-SCC*mec* III/t924. In line with previous study from Iran (12), ST239-SCC*mec* III/t037 clone is currently more frequent in our hospitals (23.3%). The review of the literature reveals that the multiresistant ST239 clone is responsible for at least 90% of HA-MRSA infections in Europe, United States, and some Asian countries, including Kuwait and Malaysia (29). Therefore, the presence of ST239-SCC*mec* III/t037 in our healthcare setting might be attributed to neighboring regions.

ST239-SCCmec III/t388 (22.5%) was the second most commonly detected MRSA clone. A similar result was reported by Ohadian Moghadam *et al* from Iran in which major universal MRSA clones were described as ST239, ST291, and ST30 (31).

On the other hand, the third most commonly detected clone was ST22-SCC*mec* IV/t790 (8.3%). This clone was associated with high resistance to mupirocin, carrying resistance genes, including *mecA*, *msr(A)*, *ant(4΄)-Ia*, *msr(B)*, *tet(M)*, and *mupA*. In our study, all ST22-SCC*mec* IV/t790 strains contained *pvl* genes. There are reports of *S. aureus* ST22 harboring *pvl* gene from Iran (12), England (33), Saudi Arabia (30), and Kuwait (34). In this regard, a study on PVL-positive MDR-MRSA isolates by Ellington *et al* (33) from England reported ST5, ST22, ST772, ST80, ST8, and ST59 strains, as later confirmed by Nadig and colleagues (35). 

Based on the findings, in clinical MRSA strains, ST15-SCC*mec* IV/t084 (7.5%) was the fourth most commonly detected clone. The low frequency of this clone has been previously reported in 16 European countries (36). PVL-carrying ST15 isolates were identified in a study by Rasigade and colleagues on 211* S. aureus *strains from 19 different countries (37). Five (4.2%) ST15-SCC*mec* IV/t084 isolates carried *pvl* genes in our study. Previously, PVL-positive ST15 was reported by Japoni-Nejad and colleagues in Iran (10). In contrast to several studies in which ST15 was reported to be prevalent among CA- and HA-MSSA isolates, all ST15 isolates belonged to MRSA strains in our study. We reported the presence of ST585-SCC*mec* III/t713 in 5% of isolates. In another study by Goudarzi *et al* the molecular features of MRSA isolates were identified, and ST585-SCC*mec* III/t713 was reported in 12% of blood samples from bacteremia patients (12). 

To sum up, the present findings showed that MRSA isolates have various genetic backgrounds in our hospitals and involve six major clones. Certain molecular types were associated with some resistance and virulence genes (e.g., *eta* with ST22-SCC*mec* IV/t790, ST15-SCC*mec* IV/t084, and ST239-SCC*mec* III/t037; *mupA* with ST15-SCC*mec* IV/t084 and ST22-SCC*mec* IV/t790; *pvl* with ST15-SCC*mec* IV/t084 and ST22-SCC*mec* IV/t790; *etb* with ST585-SCC*mec* III/t713 and ST22-SCC*mec* IV/t790). The presence of eight different *spa* types, i.e., t037, t388, t713, t924, t790, t196, t084, and t171, was also confirmed. For the first time in Iran, STs 182 and 123, as well as *spa* types t196 and t171, were detected, which might be indicative of the emergence of new clones. Further studies on other neighboring regions, focusing on the emergence of new circulating clones, are necessary to reach an overall understanding of dynamic MRSA clones in Iran and the Middle East.
